# *rac*-Methyl (3aR*,4S*,5R*,7aR*)-5,7a-bis­(acet­yloxy)-3-oxo-2-phenyl­octa­hydro-1*H*-iso­indole-4-carboxyl­ate

**DOI:** 10.1107/S1600536813025129

**Published:** 2013-09-18

**Authors:** Flavien A. A. Toze, Eugeniya V. Nikitina, Vladimir P. Zaytsev, Fedor I. Zubkov, Victor N. Khrustalev

**Affiliations:** a Department of Chemistry, University of Douala, Faculty of Sciences, PO Box 24157, Douala, Republic of , Cameroon; b Organic Chemistry Department, Peoples’ Friendship University of Russia, Miklukho-Maklaya St. 6, Moscow, 117198, Russian Federation; c X-Ray Structural Centre, A.N. Nesmeyanov Institute of Organoelement Compounds, Russian Academy of Sciences, 28 Vavilov St, B-334, Moscow 119991, Russian Federation

## Abstract

The title molecule, C_20_H_23_NO_7_, the product of nucleophilic cleavage of the 3a,6-ep­oxy bridge in 1-oxo-2-phenyl­octa­hydro-3a,6-ep­oxy­iso­indole-7-carboxyl­ate, comprises a *cis*-fused bicyclic system containing a 2-pyrrolidinone ring in an envelope conformation (with the C atom bearing the carboxyl­ate substituent as the flap) and a cyclo­hexane ring in a chair conformation. The carboxyl­ate substituent occupies the equatorial position, whereas the two acet­yloxy substituents are in axial positions. The N atom has a trigonal-planar geometry, the sum of the bond angles being 359.3 (3)°. The dihedral angle between the mean plane of the four planar atoms of the pyrrolidinone ring and the phenyl ring is 25.98 (6)°. In the crystal, mol­ecules are linked into zigzag chains along the *c-*axis direction by C—H⋯O hydrogen bonds.

## Related literature

For the synthesis of 3a,6-ep­oxy­iso­indoles by intra­molecular Diels–Alder reactions of furan, see: Vogel *et al.* (1999 ▶); Zubkov *et al.* (2005 ▶). For the synthesis of 2-phenyl­octa­hydro­iso­indoles and their analogues, see: Balthaser *et al.* (2011 ▶); Zubkov *et al.* (2011 ▶). For related compounds, see: Zubkov *et al.* (2009 ▶, 2012 ▶); Claeys *et al.* (2010 ▶).
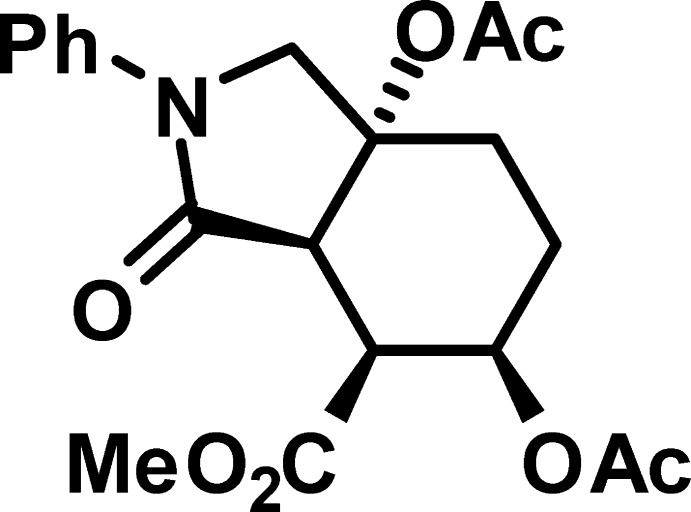


## Experimental

### 

#### Crystal data

C_20_H_23_NO_7_
*M* *_r_* = 389.39
Monoclinic, 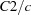

*a* = 12.3802 (7) Å
*b* = 18.3516 (10) Å
*c* = 17.3596 (9) Å
β = 102.749 (1)°
*V* = 3846.8 (4) Å^3^
*Z* = 8
Mo *K*α radiation
μ = 0.10 mm^−1^
*T* = 120 K
0.24 × 0.20 × 0.18 mm

#### Data collection

Bruker APEXII CCD diffractometer
Absorption correction: multi-scan (*SADABS*, Bruker, 2003 ▶) *T*_min_ = 0.976, *T*_max_ = 0.982
24538 measured reflections
5633 independent reflections
4521 reflections with *I* > 2σ(*I*)
*R*_int_ = 0.031

#### Refinement

*R*[*F* ^2^ > 2σ(*F* ^2^)] = 0.041
*wR*(*F* ^2^) = 0.108
*S* = 1.04
5633 reflections
256 parameters
H-atom parameters constrained
Δρ_max_ = 0.34 e Å^−3^
Δρ_min_ = −0.27 e Å^−3^

### 

Data collection: *APEX2* (Bruker, 2005 ▶); cell refinement: *SAINT* (Bruker, 2001 ▶); data reduction: *SAINT*; program(s) used to solve structure: *SHELXTL* (Sheldrick, 2008 ▶); program(s) used to refine structure: *SHELXTL*; molecular graphics: *SHELXTL*; software used to prepare material for publication: *SHELXTL*.

## Supplementary Material

Crystal structure: contains datablock(s) global, I. DOI: 10.1107/S1600536813025129/aa2096sup1.cif

Structure factors: contains datablock(s) I. DOI: 10.1107/S1600536813025129/aa2096Isup2.hkl

Supplementary material file. DOI: 10.1107/S1600536813025129/aa2096Isup3.cml

Additional supplementary materials:  crystallographic information; 3D view; checkCIF report

## Figures and Tables

**Table 1:** Hydrogen-bond geometry (Å, °)

*D*—H⋯*A*	*D*—H	H⋯*A*	*D*⋯*A*	*D*—H⋯*A*
C3*A*—H3*A*⋯O3^i^	1.00	2.55	3.4135 (13)	144
C12—H12⋯O2^ii^	0.95	2.46	3.2812 (15)	145

Symmetry codes: (i) 
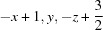
; (ii) 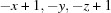.

**Fig. 1.**
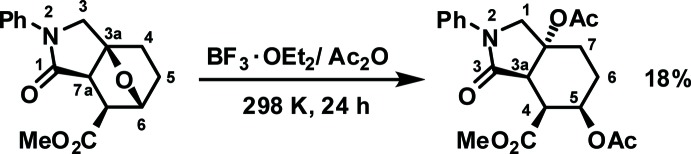
Reaction of a nucleophilic cleavage of 3a,6-epoxy bridge in 1-oxo-2-phenyloctahydro-3a,6-epoxyisoindole-7-carboxylate.

**Fig. 2.**
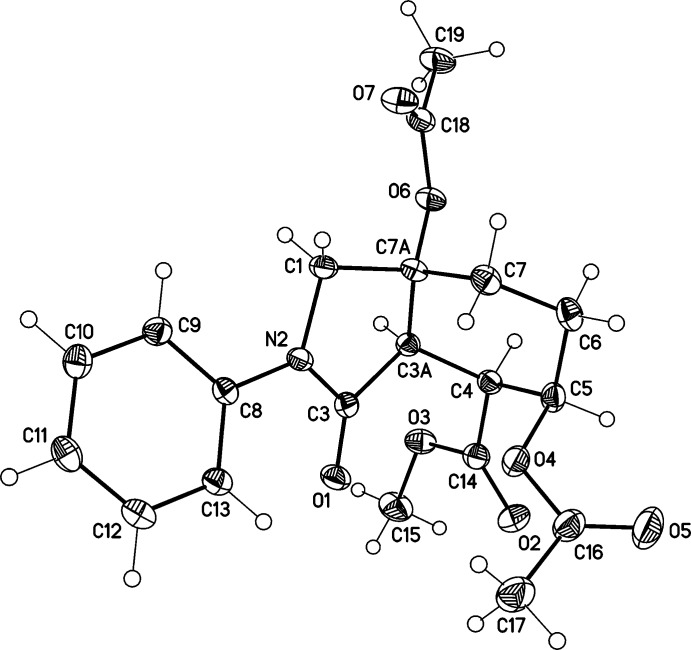
Molecular structure of the title compound. Displacement ellipsoids are shown at the 50% probability level. H atoms are presented as small spheres of arbitrary radius.

**Fig. 3.**
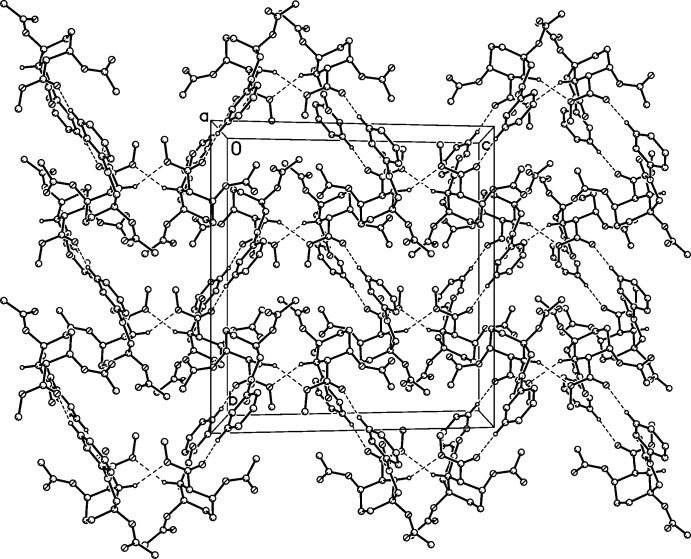
A portion of the crystal packing of the title compound demonstrating the H-bonded zigzag chains along the c axis. Dashed lines indicate the intermolecular C—H···O hydrogen bonds.

## supplementary crystallographic information

### 1. Comment

3a,6-Epoxyisoindoles, which are very easy prepared by intramolecular Diels-Alder reaction of furan (IMDAF) (Vogel *et al.*, 1999; Zubkov *et al.*, 2005), find a wide application for synthesis of various complicated natural-like molecules (Balthaser *et al.*, 2011; Zubkov *et al.*, 2011). Most of these transformations proceed *via* electrophilic or nucleophilic opening of the epoxy bridge. As a rule, the first leads to aromatic compounds, whereas the latter gives rise to perhydroisoindoles with several (three or four) asymmetric centers in mild conditions (Zubkov *et al.*, 2009, 2012; Claeys *et al.*, 2010). Stereochemistry of the nucleophilic process is hardly predictable, because it depends on mechanism of the reaction (S_N_1 or S_N_2).

This work clarifies a question concerning mechanism (S_N_2) and stereochemistry of a nucleophilic cleavage of 3a,6-epoxy bridge in 1-oxo-2-phenyloctahydro-3a,6-epoxyisoindole-7-carboxylate (Fig. 1). The structure of final product – methyl 5,7a-bis(acetyloxy)-3-oxo-2-phenyloctahydro-1*H*-isoindole- 4-carboxylate, C_20_H_23_NO_7_, was established by X-ray diffraction study.

Molecule of the title compound comprises a *cis*-fused bicyclic system containing one five-membered (2-pyrrolidinone) and one six-membered (cyclohexane) rings (Fig. 2). The five-membered ring has *envelope* conformation (the C7A carbon atom is out of the plane through the other atoms of the ring by 0.540 (2) Å), and the six-membered ring adopts *chair* conformation. The carboxylate substituent at the C4 carbon atom occupies the equatorial position, whereas the two acetyloxy substituents at the C5 and C7A carbon atoms are in the sterically unfavorable axial positions. Such disposition is explained by the direction of the nucleophilic cleavage of 3a,6-epoxy bridge in the initial 1-oxo-2-phenyloctahydro-3a,6-epoxyisoindole-7-carboxylate. The nitrogen N2 atom has a trigonal-planar geometry (sum of the bond angles is 359.3 (3)°). The dihedral angle between the planar part of the pyrrolidinone ring and phenyl ring plane is 25.98 (6)°.

The molecule of the title compound> possesses four asymmetric centers at the C3A, C4, C5 and C7A carbon atoms and can have potentially numerous diastereomers. The crystal of the title compound is racemic and consists of enantiomeric pairs with the following relative configuration of the centers: *rac*-3a*R**,4*S**,5*R**,7a*R**.

In the crystal, the molecules of the title compound are bound into the *zigzag* chains along the *c* axis by the intermolecular C—H···O hydrogen bonds (Figure 3, Table 1).

### 2. Experimental

BF_3_\ctdotEt_2_O (0.22 ml, 1.7 mmol) was added to a solution of the methyl 1-oxo-2-phenyloctahydro-3a,6-epoxyisoindole-7-carboxylate (0.2 g, 0.7 mmol) in acetic anhydride (5 ml) with stirring at room temperature during 24 h (monitoring by thin-layer chromatography). At the end of the reaction, the mixture was poured into water (50 ml), treated by aqueous sodium bicarbonate and extracted with chloroform (3 x 20 ml). The extract was dried over anhydrous magnesium sulfate. The residue was purified by crystallization from hexane – ethyl acetate to give product I (0.05 g, 0.13 mmol) as colourless solid. Yield 18%. The single-crystals of I were obtained by slow crystallization from a hexane – ethyl acetate mixture. *M*.p. = 418–419 K. IR (KBr), ν/cm^-1^: 1726, 1745 (NCO, CO_2_CH_3_, COCH_3_). ^1^H NMR (400 MHz, CDCl_3_, 293 K): *δ* = 7.54 (d, 2H, H2'(6'), *J*_2'(6'),3'(5')_ = 7.6), 7.35 (t, 2H, H3'(5'), *J*_2'(6'),3'(5')_ = *J*_4',3'(5')_ = 7.6), 7.14 (t, 1H, H4', *J*_3',4'_ = *J*_4',5'_ = 7.6), 5.59 (br. s, 1H, H5), 4.21 (d, 1H, H1A, *J*_1 A,1B_ = 10.2), 4.01 (d, 1H, H1B, *J*_1 A,1B_ = 10.2), 3.75 (s, 3H, CO_2_*Me*), 3.59 (d, 1H, H3a, *J*_3a,4_ = 5.7), 2.91 (dd, 1H, H4, *J*_4,5_ = 1.9, *J*_3a,4_ = 5.7), 2.13 (s, 3H, CO*Me*), 2.05 (s, 3H, CO*Me*), 1.57–1.66, 1.89–2.03, 2.37–2.45, (m, 4H, H6, H7). ^13^C NMR (100 MHz, CDCl_3_, 293 K): δ = 170.5, 170.2, 170.0, 168.3 (C3, 2 *x C*OCH_3_, *C*O_2_CH_3_), 138.9 (C1'), 129.0 (C3'(5')), 124.9 (C4'), 119.9 (C2'(6')), 79.0 (C7a), 65.5 (C5), 57.2 (C1), 52.0 (CO_2_*Me*), 48.3 (C3a), 40.9 (C4), 24.9, 23.9 (C6, C7), 21.2, 21.6 (2 *x* CO*Me*). Mass spectrum (EI—MS, 70 eV), *m/z* (I_r_, (%)): 389 [*M*^+^] (33), 329 (100), 287 (28), 269 (22), 242 (26), 227 (16), 210 (68), 191 (33), 182 (33), 172 (16), 163 (16), 113 (15), 105 (52), 91 (67), 80 (47), 76 (83), 59 (43), 43 (52). Anal. Calcd. for C_20_H_23_NO_7_: C, 61.69; H, 5.95; N, 3.60. Found: C, 61.49; H, 6.04; N, 3.83.

### 3. Refinement

The hydrogen atoms were placed in calculated positions with C—H = 0.95–1.00 Å and refined in the riding model with fixed isotropic displacement parameters [*U*_iso_(H) = 1.5*U*_eq_(C) for CH_3_-groups and *U*_iso_(H) = 1.2*U*_eq_(C) for the other groups].

### Crystal data

**Table:**

C_20_H_23_NO_7_	*F*(000) = 1648
*M**_r_* = 389.39	*D*_x_ = 1.345 Mg m^−^^3^
Monoclinic, *C*2/*c*	Mo *K*α radiation, λ = 0.71073 Å
Hall symbol: -C 2yc	Cell parameters from 6890 reflections
*a* = 12.3802 (7) Å	θ = 2.2–32.6°
*b* = 18.3516 (10) Å	µ = 0.10 mm^−^^1^
*c* = 17.3596 (9) Å	*T* = 120 K
β = 102.749 (1)°	Prism, colourless
*V* = 3846.8 (4) Å^3^	0.24 × 0.20 × 0.18 mm
*Z* = 8	

### Data collection

**Table:**

Bruker APEXII CCD diffractometer	5633 independent reflections
Radiation source: fine-focus sealed tube	4521 reflections with *I* > 2σ(*I*)
Graphite monochromator	*R*_int_ = 0.031
φ and ω scans	θ_max_ = 30.0°, θ_min_ = 2.0°
Absorption correction: multi-scan (*SADABS*, Bruker, 2003)	*h* = −17→17
*T*_min_ = 0.976, *T*_max_ = 0.982	*k* = −25→25
24538 measured reflections	*l* = −24→24

### Refinement

**Table:**

Refinement on *F*^2^	Primary atom site location: structure-invariant direct methods
Least-squares matrix: full	Secondary atom site location: difference Fourier map
*R*[*F*^2^ > 2σ(*F*^2^)] = 0.041	Hydrogen site location: inferred from neighbouring sites
*wR*(*F*^2^) = 0.108	H-atom parameters constrained
*S* = 1.04	*w* = 1/[σ^2^(*F*_o_^2^) + (0.0547*P*)^2^ + 1.6602*P*] where *P* = (*F*_o_^2^ + 2*F*_c_^2^)/3
5633 reflections	(Δ/σ)_max_ = 0.001
256 parameters	Δρ_max_ = 0.34 e Å^−^^3^
0 restraints	Δρ_min_ = −0.27 e Å^−^^3^

### Special details

**Table:**

Geometry. All e.s.d.'s (except the e.s.d. in the dihedral angle between two l.s. planes) are estimated using the full covariance matrix. The cell e.s.d.'s are taken into account individually in the estimation of e.s.d.'s in distances, angles and torsion angles; correlations between e.s.d.'s in cell parameters are only used when they are defined by crystal symmetry. An approximate (isotropic) treatment of cell e.s.d.'s is used for estimating e.s.d.'s involving l.s. planes.
Refinement. Refinement of *F*^2^ against ALL reflections. The weighted *R*-factor *wR* and goodness of fit *S* are based on *F*^2^, conventional *R*-factors *R* are based on *F*, with *F* set to zero for negative *F*^2^. The threshold expression of *F*^2^ > σ(*F*^2^) is used only for calculating *R*-factors(gt) *etc*. and is not relevant to the choice of reflections for refinement. *R*-factors based on *F*^2^ are statistically about twice as large as those based on *F*, and *R*- factors based on ALL data will be even larger.

### Fractional atomic coordinates and isotropic or equivalent isotropic displacement parameters (Å^2^)

**Table:**

	*x*	*y*	*z*	*U*_iso_*/*U*_eq_	
O1	0.56464 (7)	0.07894 (4)	0.60114 (5)	0.02273 (18)	
O2	0.29290 (7)	0.12128 (5)	0.54848 (6)	0.0303 (2)	
O3	0.37833 (7)	0.11721 (4)	0.67737 (5)	0.02327 (18)	
O4	0.45957 (7)	0.19171 (5)	0.47486 (5)	0.02225 (18)	
O5	0.32731 (9)	0.21367 (6)	0.36585 (6)	0.0436 (3)	
O6	0.61038 (6)	0.31985 (4)	0.69818 (5)	0.01906 (17)	
O7	0.76385 (7)	0.38200 (5)	0.68767 (5)	0.02475 (18)	
C1	0.73913 (9)	0.23138 (6)	0.65747 (7)	0.0180 (2)	
H1A	0.7920	0.2569	0.6316	0.022*	
H1B	0.7697	0.2299	0.7152	0.022*	
N2	0.71434 (7)	0.15773 (5)	0.62498 (6)	0.01699 (18)	
C3	0.60532 (9)	0.13840 (6)	0.61919 (6)	0.0167 (2)	
C3A	0.54746 (8)	0.20432 (5)	0.64506 (6)	0.01519 (19)	
H3A	0.5493	0.1979	0.7025	0.018*	
C4	0.42576 (9)	0.21450 (6)	0.60303 (6)	0.0169 (2)	
H4	0.3945	0.2518	0.6340	0.020*	
C5	0.41288 (9)	0.24521 (6)	0.52002 (6)	0.0200 (2)	
H5	0.3329	0.2536	0.4954	0.024*	
C6	0.47768 (10)	0.31571 (6)	0.52277 (7)	0.0230 (2)	
H6A	0.4495	0.3516	0.5562	0.028*	
H6B	0.4667	0.3362	0.4688	0.028*	
C7	0.60063 (10)	0.30302 (6)	0.55594 (7)	0.0207 (2)	
H7A	0.6400	0.3503	0.5590	0.025*	
H7B	0.6300	0.2712	0.5193	0.025*	
C7A	0.62497 (9)	0.26789 (5)	0.63798 (6)	0.0160 (2)	
C8	0.79927 (9)	0.11026 (6)	0.61369 (6)	0.0165 (2)	
C9	0.90772 (9)	0.12300 (6)	0.65522 (7)	0.0196 (2)	
H9	0.9234	0.1630	0.6906	0.023*	
C10	0.99269 (10)	0.07721 (6)	0.64477 (7)	0.0220 (2)	
H10	1.0662	0.0858	0.6735	0.026*	
C11	0.97091 (10)	0.01913 (6)	0.59273 (7)	0.0220 (2)	
H11	1.0291	−0.0122	0.5859	0.026*	
C12	0.86307 (10)	0.00707 (6)	0.55054 (7)	0.0214 (2)	
H12	0.8481	−0.0324	0.5144	0.026*	
C13	0.77700 (9)	0.05211 (6)	0.56061 (6)	0.0190 (2)	
H13	0.7036	0.0434	0.5316	0.023*	
C14	0.35836 (9)	0.14570 (6)	0.60423 (7)	0.0205 (2)	
C15	0.33283 (11)	0.04564 (7)	0.68386 (9)	0.0309 (3)	
H15A	0.3523	0.0296	0.7391	0.046*	
H15B	0.3633	0.0113	0.6510	0.046*	
H15C	0.2521	0.0474	0.6659	0.046*	
C16	0.40743 (10)	0.17976 (7)	0.39944 (7)	0.0265 (3)	
C17	0.46300 (12)	0.11914 (8)	0.36509 (8)	0.0348 (3)	
H17A	0.4301	0.1148	0.3085	0.052*	
H17B	0.4531	0.0733	0.3917	0.052*	
H17C	0.5422	0.1297	0.3726	0.052*	
C18	0.68623 (10)	0.37373 (6)	0.71805 (7)	0.0199 (2)	
C19	0.65958 (11)	0.42028 (7)	0.78205 (8)	0.0273 (3)	
H19A	0.7274	0.4436	0.8115	0.041*	
H19B	0.6279	0.3899	0.8180	0.041*	
H19C	0.6059	0.4578	0.7586	0.041*	

### Atomic displacement parameters (Å^2^)

**Table:**

	*U*^11^	*U*^22^	*U*^33^	*U*^12^	*U*^13^	*U*^23^
O1	0.0219 (4)	0.0148 (4)	0.0313 (4)	−0.0026 (3)	0.0054 (3)	−0.0028 (3)
O2	0.0248 (4)	0.0285 (5)	0.0336 (5)	−0.0059 (4)	−0.0025 (4)	−0.0037 (4)
O3	0.0257 (4)	0.0189 (4)	0.0263 (4)	−0.0069 (3)	0.0080 (3)	−0.0004 (3)
O4	0.0203 (4)	0.0270 (4)	0.0184 (4)	0.0050 (3)	0.0018 (3)	−0.0028 (3)
O5	0.0446 (6)	0.0527 (7)	0.0259 (5)	0.0213 (5)	−0.0089 (4)	−0.0071 (4)
O6	0.0214 (4)	0.0154 (4)	0.0221 (4)	−0.0019 (3)	0.0086 (3)	−0.0049 (3)
O7	0.0274 (4)	0.0197 (4)	0.0288 (4)	−0.0068 (3)	0.0099 (4)	−0.0040 (3)
C1	0.0179 (5)	0.0146 (5)	0.0215 (5)	−0.0019 (4)	0.0045 (4)	−0.0033 (4)
N2	0.0161 (4)	0.0130 (4)	0.0216 (4)	−0.0002 (3)	0.0037 (3)	−0.0028 (3)
C3	0.0171 (5)	0.0154 (5)	0.0170 (5)	0.0004 (4)	0.0024 (4)	0.0014 (4)
C3A	0.0159 (5)	0.0141 (4)	0.0155 (5)	−0.0009 (4)	0.0033 (4)	0.0004 (4)
C4	0.0162 (5)	0.0149 (5)	0.0197 (5)	0.0005 (4)	0.0043 (4)	−0.0010 (4)
C5	0.0189 (5)	0.0218 (5)	0.0187 (5)	0.0057 (4)	0.0026 (4)	−0.0001 (4)
C6	0.0276 (6)	0.0197 (5)	0.0219 (5)	0.0043 (4)	0.0058 (4)	0.0064 (4)
C7	0.0251 (6)	0.0176 (5)	0.0206 (5)	−0.0011 (4)	0.0076 (4)	0.0032 (4)
C7A	0.0199 (5)	0.0121 (4)	0.0168 (5)	−0.0010 (4)	0.0062 (4)	−0.0016 (4)
C8	0.0186 (5)	0.0146 (5)	0.0168 (5)	0.0016 (4)	0.0049 (4)	0.0014 (4)
C9	0.0198 (5)	0.0204 (5)	0.0184 (5)	0.0002 (4)	0.0038 (4)	−0.0022 (4)
C10	0.0193 (5)	0.0250 (6)	0.0221 (5)	0.0026 (4)	0.0054 (4)	0.0013 (4)
C11	0.0248 (6)	0.0204 (5)	0.0237 (5)	0.0050 (4)	0.0114 (4)	0.0028 (4)
C12	0.0284 (6)	0.0162 (5)	0.0215 (5)	−0.0003 (4)	0.0099 (4)	−0.0008 (4)
C13	0.0223 (5)	0.0159 (5)	0.0190 (5)	−0.0008 (4)	0.0053 (4)	−0.0003 (4)
C14	0.0160 (5)	0.0193 (5)	0.0263 (6)	0.0002 (4)	0.0053 (4)	−0.0027 (4)
C15	0.0310 (7)	0.0203 (6)	0.0432 (8)	−0.0081 (5)	0.0118 (6)	0.0019 (5)
C16	0.0265 (6)	0.0311 (6)	0.0201 (5)	0.0015 (5)	0.0012 (4)	−0.0032 (5)
C17	0.0345 (7)	0.0412 (8)	0.0265 (6)	0.0057 (6)	0.0023 (5)	−0.0109 (6)
C18	0.0236 (5)	0.0140 (5)	0.0222 (5)	−0.0007 (4)	0.0048 (4)	−0.0016 (4)
C19	0.0324 (6)	0.0205 (5)	0.0307 (6)	−0.0020 (5)	0.0104 (5)	−0.0097 (5)

### Geometric parameters (Å, º)

**Table:**

O1—C3	1.2137 (13)	C6—H6B	0.9900
O2—C14	1.2034 (14)	C7—C7A	1.5315 (15)
O3—C14	1.3446 (14)	C7—H7A	0.9900
O3—C15	1.4433 (14)	C7—H7B	0.9900
O4—C16	1.3438 (14)	C8—C9	1.3964 (15)
O4—C5	1.4536 (13)	C8—C13	1.3970 (15)
O5—C16	1.2066 (15)	C9—C10	1.3893 (15)
O6—C18	1.3548 (13)	C9—H9	0.9500
O6—C7A	1.4552 (12)	C10—C11	1.3847 (17)
O7—C18	1.2034 (14)	C10—H10	0.9500
C1—N2	1.4705 (13)	C11—C12	1.3915 (17)
C1—C7A	1.5329 (15)	C11—H11	0.9500
C1—H1A	0.9900	C12—C13	1.3897 (16)
C1—H1B	0.9900	C12—H12	0.9500
N2—C3	1.3776 (14)	C13—H13	0.9500
N2—C8	1.4121 (13)	C15—H15A	0.9800
C3—C3A	1.5232 (15)	C15—H15B	0.9800
C3A—C7A	1.5326 (14)	C15—H15C	0.9800
C3A—C4	1.5336 (15)	C16—C17	1.4992 (18)
C3A—H3A	1.0000	C17—H17A	0.9800
C4—C14	1.5161 (15)	C17—H17B	0.9800
C4—C5	1.5226 (15)	C17—H17C	0.9800
C4—H4	1.0000	C18—C19	1.4949 (16)
C5—C6	1.5176 (17)	C19—H19A	0.9800
C5—H5	1.0000	C19—H19B	0.9800
C6—C7	1.5218 (16)	C19—H19C	0.9800
C6—H6A	0.9900		
			
C14—O3—C15	115.72 (10)	O6—C7A—C1	112.49 (9)
C16—O4—C5	118.23 (9)	C7—C7A—C1	111.83 (9)
C18—O6—C7A	118.17 (8)	C3A—C7A—C1	102.28 (8)
N2—C1—C7A	102.29 (8)	C9—C8—C13	119.72 (10)
N2—C1—H1A	111.3	C9—C8—N2	119.05 (9)
C7A—C1—H1A	111.3	C13—C8—N2	121.21 (10)
N2—C1—H1B	111.3	C10—C9—C8	120.05 (10)
C7A—C1—H1B	111.3	C10—C9—H9	120.0
H1A—C1—H1B	109.2	C8—C9—H9	120.0
C3—N2—C8	125.34 (9)	C11—C10—C9	120.45 (11)
C3—N2—C1	112.51 (9)	C11—C10—H10	119.8
C8—N2—C1	121.44 (9)	C9—C10—H10	119.8
O1—C3—N2	126.48 (10)	C10—C11—C12	119.46 (10)
O1—C3—C3A	126.50 (10)	C10—C11—H11	120.3
N2—C3—C3A	106.91 (9)	C12—C11—H11	120.3
C3—C3A—C7A	103.73 (8)	C13—C12—C11	120.83 (10)
C3—C3A—C4	115.66 (9)	C13—C12—H12	119.6
C7A—C3A—C4	115.84 (8)	C11—C12—H12	119.6
C3—C3A—H3A	107.0	C12—C13—C8	119.48 (10)
C7A—C3A—H3A	107.0	C12—C13—H13	120.3
C4—C3A—H3A	107.0	C8—C13—H13	120.3
C14—C4—C5	112.22 (9)	O2—C14—O3	124.43 (11)
C14—C4—C3A	112.21 (9)	O2—C14—C4	125.04 (11)
C5—C4—C3A	112.46 (9)	O3—C14—C4	110.47 (9)
C14—C4—H4	106.5	O3—C15—H15A	109.5
C5—C4—H4	106.5	O3—C15—H15B	109.5
C3A—C4—H4	106.5	H15A—C15—H15B	109.5
O4—C5—C6	108.81 (9)	O3—C15—H15C	109.5
O4—C5—C4	106.82 (9)	H15A—C15—H15C	109.5
C6—C5—C4	109.97 (9)	H15B—C15—H15C	109.5
O4—C5—H5	110.4	O5—C16—O4	123.61 (12)
C6—C5—H5	110.4	O5—C16—C17	126.23 (12)
C4—C5—H5	110.4	O4—C16—C17	110.16 (10)
C5—C6—C7	111.04 (9)	C16—C17—H17A	109.5
C5—C6—H6A	109.4	C16—C17—H17B	109.5
C7—C6—H6A	109.4	H17A—C17—H17B	109.5
C5—C6—H6B	109.4	C16—C17—H17C	109.5
C7—C6—H6B	109.4	H17A—C17—H17C	109.5
H6A—C6—H6B	108.0	H17B—C17—H17C	109.5
C6—C7—C7A	113.04 (9)	O7—C18—O6	123.80 (10)
C6—C7—H7A	109.0	O7—C18—C19	125.60 (11)
C7A—C7—H7A	109.0	O6—C18—C19	110.60 (10)
C6—C7—H7B	109.0	C18—C19—H19A	109.5
C7A—C7—H7B	109.0	C18—C19—H19B	109.5
H7A—C7—H7B	107.8	H19A—C19—H19B	109.5
O6—C7A—C7	111.22 (8)	C18—C19—H19C	109.5
O6—C7A—C3A	105.19 (8)	H19A—C19—H19C	109.5
C7—C7A—C3A	113.38 (9)	H19B—C19—H19C	109.5
			
C7A—C1—N2—C3	−23.81 (11)	C4—C3A—C7A—O6	82.25 (10)
C7A—C1—N2—C8	165.34 (9)	C3—C3A—C7A—C7	88.38 (10)
C8—N2—C3—O1	−2.61 (18)	C4—C3A—C7A—C7	−39.48 (12)
C1—N2—C3—O1	−173.04 (11)	C3—C3A—C7A—C1	−32.19 (10)
C8—N2—C3—C3A	173.69 (9)	C4—C3A—C7A—C1	−160.05 (9)
C1—N2—C3—C3A	3.26 (12)	N2—C1—C7A—O6	145.93 (8)
O1—C3—C3A—C7A	−164.92 (11)	N2—C1—C7A—C7	−88.07 (10)
N2—C3—C3A—C7A	18.78 (11)	N2—C1—C7A—C3A	33.57 (10)
O1—C3—C3A—C4	−36.95 (15)	C3—N2—C8—C9	−149.57 (11)
N2—C3—C3A—C4	146.75 (9)	C1—N2—C8—C9	20.06 (15)
C3—C3A—C4—C14	51.02 (12)	C3—N2—C8—C13	31.90 (16)
C7A—C3A—C4—C14	172.71 (9)	C1—N2—C8—C13	−158.47 (10)
C3—C3A—C4—C5	−76.63 (11)	C13—C8—C9—C10	−1.15 (16)
C7A—C3A—C4—C5	45.06 (12)	N2—C8—C9—C10	−179.70 (10)
C16—O4—C5—C6	−100.66 (11)	C8—C9—C10—C11	0.60 (17)
C16—O4—C5—C4	140.66 (10)	C9—C10—C11—C12	0.30 (17)
C14—C4—C5—O4	−64.68 (11)	C10—C11—C12—C13	−0.66 (17)
C3A—C4—C5—O4	62.96 (11)	C11—C12—C13—C8	0.11 (16)
C14—C4—C5—C6	177.40 (9)	C9—C8—C13—C12	0.79 (16)
C3A—C4—C5—C6	−54.96 (12)	N2—C8—C13—C12	179.31 (10)
O4—C5—C6—C7	−55.63 (12)	C15—O3—C14—O2	12.04 (17)
C4—C5—C6—C7	61.05 (12)	C15—O3—C14—C4	−170.57 (9)
C5—C6—C7—C7A	−56.34 (13)	C5—C4—C14—O2	−7.30 (16)
C18—O6—C7A—C7	−71.68 (12)	C3A—C4—C14—O2	−135.08 (12)
C18—O6—C7A—C3A	165.19 (9)	C5—C4—C14—O3	175.33 (9)
C18—O6—C7A—C1	54.65 (12)	C3A—C4—C14—O3	47.55 (12)
C6—C7—C7A—O6	−73.57 (11)	C5—O4—C16—O5	4.05 (19)
C6—C7—C7A—C3A	44.73 (12)	C5—O4—C16—C17	−175.72 (11)
C6—C7—C7A—C1	159.74 (9)	C7A—O6—C18—O7	1.55 (16)
C3—C3A—C7A—O6	−149.89 (8)	C7A—O6—C18—C19	−178.41 (9)

### Hydrogen-bond geometry (Å, º)

**Table:**

*D*—H···*A*	*D*—H	H···*A*	*D*···*A*	*D*—H···*A*
C3*A*—H3*A*···O3^i^	1.00	2.55	3.4135 (13)	144
C12—H12···O2^ii^	0.95	2.46	3.2812 (15)	145

Symmetry codes: (i) −*x*+1, *y*, −*z*+3/2; (ii) −*x*+1, −*y*, −*z*+1.

## References

[bb1] Balthaser, B. R., Maloney, M. C., Beeler, A. B., Porco, J. A. Jr & Snyder, J. K. (2011). *Nat. Chem.* **3**, 969–973. 10.1038/nchem.1178 PMC3254213 22213919

[bb2] Bruker (2001). *SAINT* Bruker AXS Inc., Madison, Wisconsin, USA.

[bb3] Bruker (2003). *SADABS* Bruker AXS Inc., Madison, Wisconsin, USA.

[bb4] Bruker (2005). *APEX2* Bruker AXS Inc., Madison, Wisconsin, USA.

[bb5] Claeys, D. D., Stevens, C. V., Roman, B. I., Caveye, P. van D., Waroquier, M. & Speybroeck, V. V. (2010). *Org. Biomol. Chem.* **8**, 3644–3654. 10.1039/c002926b 20544085

[bb6] Sheldrick, G. M. (2008). *Acta Cryst.* A**64**, 112–122. 10.1107/S0108767307043930 18156677

[bb7] Vogel, P., Cossy, J., Plumet, J. & Arjona, O. (1999). *Tetrahedron*, **55**, 13521–13642.

[bb8] Zubkov, F. I., Ershova, J. D., Orlova, A. A., Zaytsev, V. P., Nikitina, E. V., Peregudov, A. S., Gurbanov, A. V., Borisov, R. S., Khrustalev, V. N., Maharramov, A. M. & Varlamov, A. V. (2009). *Tetrahedron*, **65**, 3789–3803.

[bb9] Zubkov, F. I., Nikitina, E. V. & Varlamov, A. V. (2005). *Russ. Chem. Rev.* **74**, 639–669.

[bb10] Zubkov, F. I., Zaytsev, V. P., Nikitina, E. V., Boltukhina, E. V., Varlamov, A. V., Khrustalev, V. N. & Gozun, S. V. (2011). *Tetrahedron*, **67**, 9148–9163.

[bb11] Zubkov, F. I., Zaytsev, V. P., Puzikova, E. S., Nikitina, E. V., Varlamov, A. V., Khrustalev, V. N. & Novikov, R. A. (2012). *Chem. Heterocycl. Compd*, **48**, 514–524.

